# Cerebral small vessel disease was associated with the prognosis in ischemic stroke with atrial fibrillation

**DOI:** 10.1111/cns.70052

**Published:** 2024-10-21

**Authors:** Yicong Wang, Hang Li, Yuesong Pan, Mengxing Wang, Xiaoling Liao, Yingying Yang, Weiqi Chen, Xia Meng, Yongjun Wang, Yilong Wang

**Affiliations:** ^1^ Department of Neurology, Beijing Tiantan Hospital Capital Medical University Beijing China; ^2^ China National Clinical Research Center for Neurological Diseases Beijing China; ^3^ Department of Geriatrics Affiliated Dalian Friendship Hospital of Dalian Medical University Dalian China; ^4^ Advanced Innovation Center for Human Brain Protection, Capital Medical University Beijing China; ^5^ Research Unit of Artificial Intelligence in Cerebrovascular Disease Chinese Academy of Medical Sciences Beijing China; ^6^ Center for Excellence in Brain Science and Intelligence Technology Chinese Academy of Sciences Shanghai China; ^7^ Chinese Institute for Brain Research Beijing China; ^8^ Beijing Key Laboratory of Translational Medicine for Cerebrovascular Disease Beijing China

**Keywords:** atrial fibrillation, CSVD, ischemic stroke

## Abstract

**Background:**

The purpose of this study was to explore the relationship between atrial fibrillation (AF), cerebral small vessel disease (CSVD), and ischemic stroke.

**Methods:**

Data were extracted from China's Third National Stroke Registry (CNSR‐III), which registered 15,166 patients in China. A total of 12,180 ischemic stroke patients were included excluding those diagnosed with TIA or without MRI. Logistic regression was to explore the relationship between AF, CSVD, and poor functional outcomes at 12‐month follow‐up. Cox regression is to explore AF, CSVD, and stroke recurrence as well as all‐cause mortality at 12‐month follow‐up.

**Results:**

The average age was 62.40 ± 11.22 years old, and 8362 (68.65%) were men at baseline. Patients with AF had an increased risk of stroke recurrence and all‐cause mortality at 12‐month follow‐up. Those with AF and CSVD imaging such as lacunes, white matter hyperintensity (WMH), and the presence of cerebral microbleeds (CMBs) had an increased risk of poor prognosis. And those with both AF and CSVD burden had an increased risk of worse prognosis at 12‐month follow‐up.

**Conclusion:**

Among Chinese patients with acute ischemic stroke, those with AF were associated with a higher risk of 12‐month mortality and stroke recurrence. When AF was combined with some CSVD imaging features such as lacunes, WMH, presence of CMBs or burdens, the 12‐month poor prognosis worsened.

## Graphical abstract

The 12,180 patients were from China's Third National Stroke Registry. Patients with AF had an increased risk of stroke recurrence and all‐cause mortality at 12 months. Those with AF and CSVD such as lacunes, WMH, and CMBs had an increased risk of poor prognosis. Those with both AF and CSVD burden had an increased risk of worse prognosis at 12 months.

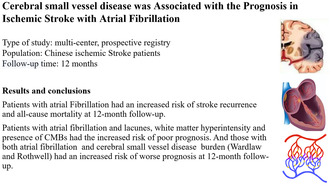

## 1 INTRODUCTION

Stroke is an important cause of death and disability all over the world, with 70%–80% of cases being ischemic stroke.^1^, ^2^ Atrial fibrillation (AF) is the most common clinically relevant cardiac arrhythmia, characterized by an irregular heart rhythm that can lead to reduced cerebral blood flow and embolic events.^3^ Compared to other etiologies of ischemic stroke, it is associated with higher severity, poorer prognosis, and higher recurrence rates.^4^, ^5^

In recent years, it has also been discovered that AF was related to the presence of asymptomatic cerebral small vessel disease (CSVD) on neuroimaging in embolic stroke patients and birth cohort.^6^, ^7^, ^8^ And in individuals with AF who did not have a history of stroke, there was an association between CSVD imaging markers and the occurrence of cognitive impairment.^9^, ^10^ CSVD was a comprehensive syndrome involving clinical, imaging, and pathological manifestations.^11^ However. the exact mechanism of CSVD was still unclear which was believed to be associated with aging, endothelial dysfunction, inflammation, and hypoperfusion.^12^, ^13^ Imaging of CSVD included lacunes, white matter hyperintensity (WMH), perivascular space (PVS), cerebral microbleeds (CMBs), and so on.^14^ It has been found that CSVD has an impact on the prognosis of ischemic stroke, such as the influence of CMBs and WMH on the prognosis of reperfusion therapy.^15^, ^16^ However, there is currently a lack of long‐term follow‐up studies on the outcomes of prognostic impact of AF and CSVD in ischemic stroke patients.

Based on the data from the China National Stroke Registry III study, we comprehensively evaluated the relationship between AF, CSVD, and 12‐month outcome of ischemic stroke in Chinese.

## 2 METHODS

### 2.1 Study design and population

Data for this study came from the Chinese National Stroke Registry III (CNSR‐III), a national, multi‐center, prospective registry of Chinese patients with transient ischemic attacks (TIA) and ischemic stroke.^17^ The registry included 15,166 patients aged 18 years and above with ischemic stroke or TIA from 201 hospitals in 22 provinces and four municipalities from August 2015 to March 2018. The time from symptom onset to enrollment should be within 7 days. The study was conducted in accordance with the Helsinki Declaration (2013 revision). The research protocol of CNSR‐III was approved by the ethics committee of Beijing Tiantan Hospital, Capital Medical University (IRB approval number: KY2015‐001‐01) and all participating centers. The diagnosis of acute ischemic stroke was confirmed by magnetic resonance imaging (MRI) or computed tomography (CT) scan according to the World Health Organization criteria.^18^

In this study, patients without MRI imaging data and those diagnosed with TIA were excluded from the analysis. In the end, a total of 12,180 ischemic stroke patients with complete data were included in the study.

### 2.2 Baseline data collection

Baseline data were collected through face‐to‐face interviews conducted by trained research personnel. The baseline data included demographic information, risk factors (hypertension, diabetes, dyslipidemia, previous ischemic stroke or TIA, precious smoking and drinking history), concomitant medication (antihypertensive drugs, lipid‐lowering drugs, antidiabetic drugs, antiplatelet drugs, and anticoagulants), laboratory test results and modified Rankin Scale (mRS) at discharge. Body mass index (BMI) was calculated as weight (kilograms) divided by height squared (m^2^). The diagnosis of atrial fibrillation was based on a confirmed medical history, electrocardiogram, or long‐term electrocardiogram monitoring. The National Institutes of Health Stroke Scale (NIHSS) was used to assess the severity of stroke.

### 2.3 Blood sample collection and laboratory tests

Blood samples were collected after admission and transported to Beijing Tiantan Hospital through the cold chain and they were stored in a − 80°C freezer in the central laboratory. Total cholesterol (TC), high‐density lipoprotein cholesterol (HDL‐C), low‐density lipoprotein cholesterol (LDL‐C), fasting glucose, and white blood cells (WBCs) were measured. All tests were performed by laboratory personnel who were blinded to the clinical information.

### 2.4 Cerebral small vessel disease imaging evaluation

The CSVD imaging evaluation in this study was also performed using brain MRI, including T1‐weighted (T1w), T2‐weighted (T2w), fluid‐attenuated inversion recovery (FLAIR), diffusion‐weighted imaging (DWI), and susceptibility‐weighted imaging (SWI) sequences. The imaging data were collected in digital format from each center and blindly reviewed by trained raters for CSVD imaging features.^14^ WMHs were defined as increased brightness in the white matter on T2 images. Both periventricular WMH and deep WMH were evaluated with Fazekas rating scale.^19^ Lacunes were defined as fluid‐filled cavities (with a signal like cerebrospinal fluid) with a diameter of 3 to 15 mm and a round or oval shape. CMBs were defined as round or oval hypointense lesions with a diameter of 2 to 10 mm on gradient‐recalled echo or susceptibility‐weighted images. Perivascular spaces were defined as small punctate (<3 mm) or linear hyperintensities on T2 images, and perivascular spaces in the basal ganglia were graded using the semiquantitative rating scale developed by the Edinburgh group.^20^ The assessment of each CSVD imaging was completed by two well‐trained raters, and in case of inconsistent results, another senior neurologist who was blinded to the initial results evaluated the images. The kappa coefficient of CSVD markers on brain MRI between raters were as follows: 0.82 for lacune, 0.88 for Fazekas scale of WMH, 0.89 for the severity of PVS, 0.85 for the presence of CMB.

The total CSVD burden score, designed by the Wardlaw group, was graded from 0 to 4.^21^ One point was included the presence of lacunes, WMH burden (periventricular WMH grade 3 or deep WMH grade 2–3), presence of CMBs, and moderate‐to‐severe basal ganglia PVS (BG‐PVS) (*N* > 10). The modified total CSVD burden score, designed by the Rothwell group, was graded from 0 to 6.^22^ One point was allocated for the presence of lacunes, CMBs burden (*N* = 1–4), severe basal ganglia PVS (*N* >20), and modified WMH burden (total periventricular + subcortical WMH grade 3–4). Two points were allocated for CMBs burden (*N* ≥5) and modified WMH burden (total periventricular + subcortical WMH grade 5–6). In the patient population included in this study, no severe basal ganglia PVS (*N* > 20) was found.

### 2.5 Follow‐up and events

Data from telephone interviews conducted at 1 year were used for follow‐up. During follow‐up, deaths, stroke recurrence, and cardiovascular events were recorded. Death referred to all‐cause mortality. Stroke recurrence includes ischemic stroke and hemorrhagic stroke. The poor functional outcomes were defined as the mRS score of 3–6. All kinds of events during follow‐up were confirmed by hospitals. If the suspected events such as stroke recurrence did not result in hospitalization, they would be adjudicated by the independent endpoint adjudication committee. Each death case was confirmed by death certificates from hospitals or local civil registration offices.

### 2.6 Statistical analysis

Continuous variables were described with mean as well as standard deviation or median as well as interquartile range (IQR), while categorical variables were described with percentages. Continuous variables were compared with t‐test and Wilcoxon rank‐sum test, while categorical variables were compared with chi‐square test or Fisher's exact test. Logistic regression analysis was also used to calculate the relationship between AF and poor functional outcomes at 12‐month follow‐up with odds ratios (ORs) as well as 95% confidence intervals (CIs). Cox regression analysis was used to calculate the relationship between AF and stroke recurrence and all‐cause mortality at 12‐month follow‐up with hazard ratios (HRs) as well as 95% CIs. Similarly, logistic and cox regression analyses were performed to assess the relationship between AF combined with different CSVD imaging and burdens and stroke outcomes at 12‐month follow‐up. Model 1 was adjusted for age, gender, and NIHSS. Model 2 was adjusted for age, gender, NIHSS, and mRS score. Model 3 was adjusted for age, gender, NIHSS, mRS, and risk factors (hypertension, diabetes, dyslipidemia, previous smoking, and previous drinking). All data analyses were performed using SAS software version 9.4 (SAS Institute, Inc, Cary, NC). *p* value of less than 0.05 was considered to indicate statistical significance.

## 3 RESULTS

### 3.1 Baseline characteristics of patients

**FIGURE 1:**
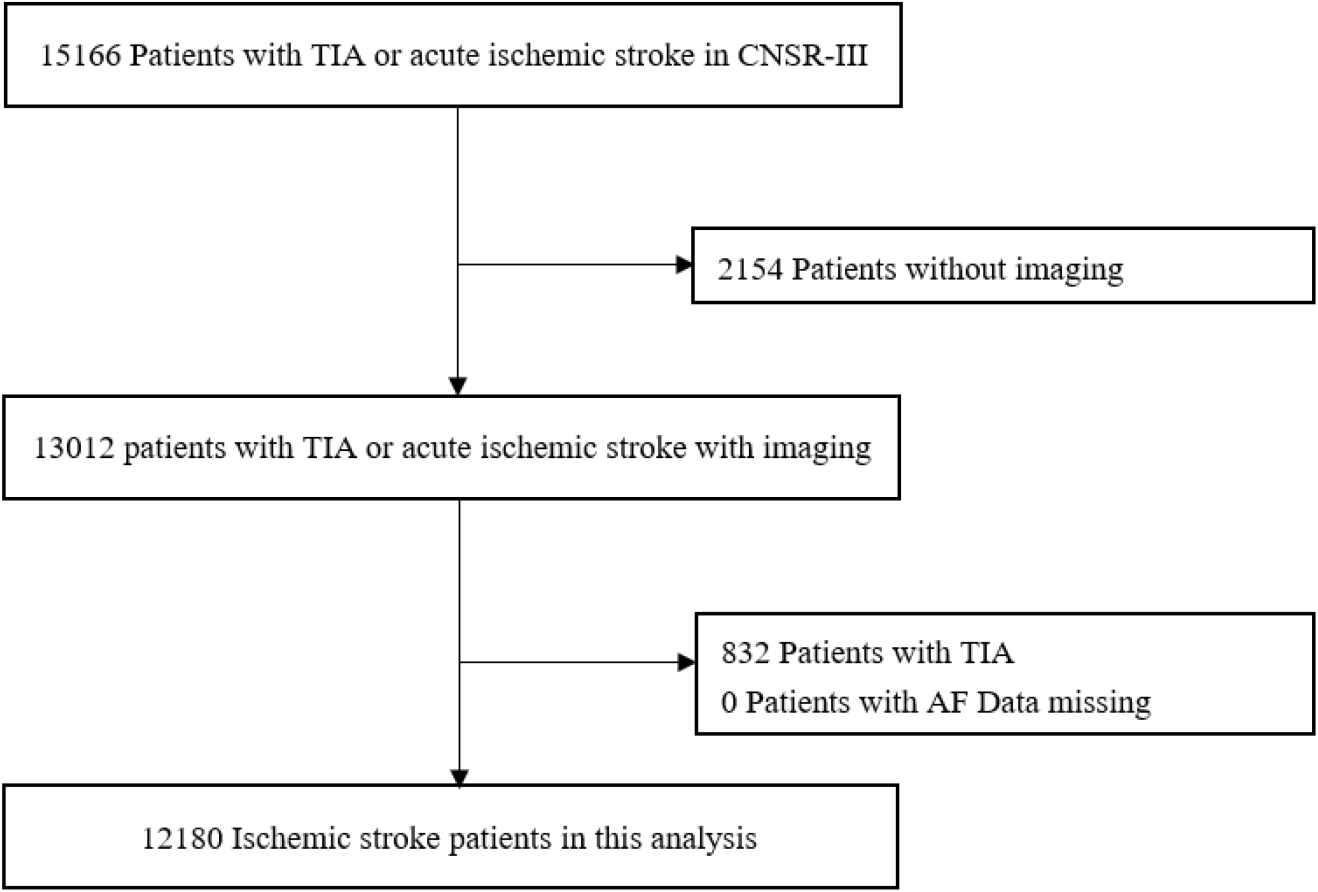
Flowchart of the study. AF, atrial fibrillation; TIA, transient ischemic attack.

The flowchart of patients included in this study is shown in Figure 1. This study included 12,180 patients with acute ischemic stroke, excluding those diagnosed with TIA and without MRI. Baseline clinical characteristics are shown in Table 1. The average patient age was 62.40 ± 11.22 years old (males, 68.65%). Compared to ischemic stroke patients without AF, those with AF were found to be older and have higher NIHSS. They also had a higher proportion of patients taking lipid‐lowering, antiplatelet, and anticoagulant drugs. Additionally, patients with atrial fibrillation had a lower proportion of males and lower proportions of hypertension, diabetes, precious history of ischemic stroke or TIA, previous smoking and drinking. In terms of laboratory, patients with atrial fibrillation had higher levels of HDL‐C and WBC, while TC levels were lower.

**TABLE 1:** Baseline characteristics of ischemic stroke patients with atrial fibrillation.

Characteristics	Total	Atrial fibrillation		
		Absence	Presence	*p* value
*N*	12,180	11,380	800	
Age, mean ± SD	62.40 ± 11.22	61.84 ± 11.09	70.34 ± 9.84	<0.001
Male, *n* (%)	8362 (68.65)	7872 (69.17)	490 (61.25)	<0.001
Admission NIHSS	4.38 ± 4.10	4.22 ± 3.88	6.55 ± 6.05	<0.001
Preadmission mRS	0.43 ± 0.88	0.42 ± 0.87	0.50 ± 1.00	0.07
Risk factors, *n* (%)				
Hypertension	7675 (63.01)	7201 (63.28)	474 (59.25)	0.02
Diabetes mellitus	2812 (23.09)	2673 (23.49)	139 (17.38)	<0.001
Dyslipidemia	923 (7.58)	876 (7.70)	47 (5.88)	0.06
Previous ischemic stroke or TIA	257 (2.11)	248 (2.18)	9 (1.13)	0.02
Previous smoking	3897 (32.00)	3738 (32.85)	159 (19.88)	<0.001
Previous drinking	1733 (14.23)	1642 (14.43)	91 (11.38)	0.02
Concomitant medication, *n* (%)				
Antihypertensive drugs	5460 (44.83)	5080 (44.64)	380 (47.50)	0.12
Lipid‐lowering drugs	1251 (10.27)	1135 (9.97)	116 (14.50)	<0.001
Antidiabetic drugs	2220 (18.23)	2111 (18.55)	109 (13.63)	<0.001
Antiplatelet drugs	1994 (16.37)	1777 (15.62)	217 (27.13)	<0.001
Anticoagulants	99 (0.81)	37 (0.33)	62 (7.75)	<0.001
Laboratory test results, median (IQR)				
LDL‐C, mmol/L	2.32 (1.73–2.99)	2.32 (1.73–2.99)	2.25 (1.71–2.97)	0.46
HDL‐C, mmol/L	0.93 (0.77–1.11)	0.93 (0.77–1.11)	0.99 (0.80–1.20)	<0.001
TC, mmol/L	3.97 (3.31–4.72)	3.98 (3.32–4.73)	3.78 (3.16–4.52)	<0.001
Fasting glucose, mmol/L	5.52 (4.90–6.93)	5.52 (4.90–6.97)	5.48 (4.82–6.64)	0.09
WBC, /L	6.92 (5.74–8.45)	6.90 (5.73–8.43)	7.18 (5.81–8.66)	0.02
CHA2DS2‐VASc score	92 (0.76)	73 (0.64)	19 (2.38)	0.61
CSVD burden (Wardlaw)	6718 (55.16)	6299 (55.35)	419 (52.38)	0.33
Modified CSVD burden (Rothwell)	5140 (42.20)	4819 (42.35)	325 (40.63)	0.46

Abbreviations: CSVD, cerebral small vessel disease; HDL, high‐density lipoprotein; LDL, low‐density lipoprotein; TC, total cholesterol; TIA, transient ischemic attack; WBC, white blood cell.

### 3.2 Associations between atrial fibrillation and 12‐month outcomes

The association between AF and 12‐month outcomes is shown in Table 2. After adjusting for age, sex, NIHSS, mRS, and risk factors (hypertension, diabetes mellitus, dyslipidemia, previous ischemic stroke or TIA) in model 3, patients with AF had an increased risk of stroke recurrence (HR 1.26, 95% CI 1.02–1.54, *p* = 0.03) and all‐cause mortality (HR 1.98, 95% CI 1.53–2.57, *p*<0.001) at 12‐month follow‐up. However, no significant increase in the risk of poor functional outcome at 12‐month follow‐up was observed in patients with AF (OR 1.06, 95% CI 0.90–1.25, *p* = 0.48) (Table 2).

**TABLE 2:** Association between atrial fibrillation and 12‐month outcomes.

	AF	Events (%)	Model 1			Model 2			Model 3		
			HR/OR	95% CI	*p*	HR/OR	95% CI	*p*	HR/OR	95% CI	*p*
Stroke recurrence	No AF	1094 (9.61)	Ref.	Ref.		Ref.	Ref.		Ref.	Ref.	
	AF	107 (13.38)	1.23	1.00–1.51	0.0495	1.23	1.003–1.51	0.046	1.26	1.02–1.54	0.03
All‐cause mortality	No AF	306 (2.69)	Ref.	Ref.		Ref.	Ref.		Ref.	Ref.	
	AF	85 (10.63)	1.89	1.46–2.45	<0.001	1.93	1.49–2.50	<0.001	1.98	1.53–2.57	<0.001
Poor functional outcome	No AF	6693 (58.81)	Ref.	Ref.		Ref.	Ref.		Ref.	Ref.	
	AF	557 (69.63)	1.03	0.87–1.22	0.71	1.03	0.88–1.22	0.69	1.06	0.90–1.25	0.48

*Note*: Model 1: adjusted for age, sex, and NIHSS; Model 2: adjusted for age, sex, NIHSS, and mRS; Model 3: adjusted for age, sex, NIHSS, mRS, and risk factors (hypertension, diabetes mellitus, dyslipidemia, previous ischemic stroke or TIA).

Abbreviation: AF, atrial fibrillation.

### 3.3 Associations between atrial fibrillation, CSVD, and 12‐month outcomes

Compared to ischemic stroke patients without AF, those with AF had an increased risk of stroke recurrence and death at 12‐month follow‐up. Further results based on CSVD imaging phenotypes revealed that ischemic stroke patients with both AF and lacunes (HR 1.41, 95% CI 1.05–1.90, *p* = 0.02), WMH (WMH burden: HR 1.56, 95% CI 1.18–2.06, *p* = 0.002; modified WMH burden: HR 1.25, 95% CI 1.01–1.54, *p*= 0.04) and presence of CMBs (HR 1.83, 95% CI 1.14–2.92, *p* = 0.01) had an increased risk of stroke recurrence at 12‐month follow‐up. Similarly, ischemic stroke patients with both AF and lacunes (HR 1.72, 95% CI 1.19–2.50, *p* = 0.004), WMH (WMH burden: HR 1.97, 95% CI 1.41–2.76, *p* < 0.001; modified WMH burden: HR 1.95, 95% CI 1.50–2.54, *p*< 0.001) and presence of CMBs (HR 2.56, 95% CI 1.45–4.50, *p* = 0.001) had an increased risk of all‐cause mortality at 12‐month follow‐up. Ischemic stroke patients with both AF and lacunes (OR 1.45, 95% CI 1.09–1.92, *p* = 0.01) had an increased risk of poor functional outcome at 12‐month follow‐up. However, no other significant increase in the risk of poor functional outcomes was observed in ischemic stroke patients with both AF and CSVD burdens (Table 3).

**TABLE 3:** Association between atrial fibrillation and CSVD and 12‐month outcomes.

	Atrial fibrillation and CSVD	Events (%)	Model 1			Model 2			Model 3		
			HR/OR	95% CI	*p*	HR/OR	95% CI	*p*	HR/OR	95% CI	*p*
Stroke recurrence	No AF	1094 (9.61)	Ref.	Ref.		Ref.	Ref.		Ref.	Ref.	
	AF	107 (13.38)	1.23	1.00–1.51	0.05	1.23	1.00–1.51	0.05	1.26	1.02–1.54	0.03
	AF with lacunes	48 (15.48)	1.42	1.06–1.91	0.02	1.42	1.06–1.91	0.02	1.41	1.05–1.90	0.02
	AF with WMH burden	56 (16.92)	1.55	1.17–2.05	0.002	1.55	1.17–2.05	0.002	1.56	1.18–2.06	0.002
	AF with modified WMH burden	104 (13.32)	1.22	0.99–1.50	0.06	1.22	0.99–1.51	0.06	1.25	1.01–1.54	0.04
	AF with PVS in basal ganglia (moderate‐to‐severe)	2 (28.57)	‐	‐	‐	‐	‐	‐	‐	‐	‐
	AF with CMBs burden	45 (13.60)	1.24	0.92–1.68	0.16	1.25	0.92–1.69	0.15	1.29	0.95–1.74	0.10
	AF with presence of CMBs	18 (18.18)	1.79	1.12–2.87	0.01	1.80	1.13–2.88	0.01	1.83	1.14–2.92	0.01
	AF with CSVD (Wardlaw)	17 (15.18)	1.37	0.85–2.23	0.20	1.39	0.85–2.25	0.19	1.46	0.90–2.37	0.12
	AF with CSVD (Rothwell)	15 (15.00)	1.33	0.80–2.22	0.28	1.34	0.80–2.25	0.26	1.38	0.83–2.31	0.22
All‐cause mortality	No AF	306 (2.69)	Ref.	Ref.		Ref.	Ref.		Ref.	Ref.	
	AF	85 (10.63)	1.89	1.46–2.45	<0.001	1.93	1.49–2.50	<0.001	1.98	1.53–2.57	<0.001
	AF with lacunes	34 (10.97)	1.70	1.17–2.47	0.006	1.75	1.20–2.53	0.003	1.72	1.19–2.50	0.004
	AF with WMH burden	43 (12.99)	1.91	1.37–2.68	<0.001	1.95	1.39–2.73	<0.001	1.97	1.41–2.76	<0.001
	AF with modified WMH burden	82 (10.50)	1.85	1.42–2.41	<0.001	1.90	1.46–2.47	<0.001	1.95	1.50–2.54	<0.001
	AF with PVS in basal ganglia (moderate‐to‐severe)	2 (28.57)	‐	‐	‐	‐	‐	‐	‐	‐	‐
	AF with CMBs burden	24 (7.25)	1.25	0.81–1.91	0.31	1.25	0.82–1.92	0.31	1.35	0.88–2.07	0.17
	AF with presence of CMBs	13 (13.13)	2.49	1.42–4.37	0.002	2.56	1.46–4.49	0.001	2.56	1.45–4.50	0.001
	AF with CSVD (Wardlaw)	13 (11.61)	1.81	1.03–3.20	0.04	1.91	1.09–3.37	0.02	2.10	1.19–3.71	0.01
	AF with CSVD (Rothwell)	4 (4.00)	‐	‐	‐	‐	‐	‐	‐	‐	‐
Poor functional outcome	No AF	6693 (58.81)	Ref.	Ref.		Ref.	Ref.		Ref.	Ref.	
	AF	557 (69.63)	1.03	0.87–1.22	0.71	1.03	0.88–1.22	0.69	1.06	0.90–1.25	0.48
	AF with lacunes	238 (76.77)	1.46	1.11–1.93	0.01	1.44	1.09–1.90	0.01	1.45	1.09–1.92	0.01
	AF with WMH burden	237 (71.60)	1.01	0.78–1.30	0.96	1.00	0.77–1.29	0.10	1.01	0.78–1.31	0.91
	AF with modified WMH burden	542 (69.40)	1.02	0.86–1.20	0.85	1.02	0.86–1.21	0.79	1.05	0.89–1.24	0.57
	AF with PVS in basal AF with ganglia (moderate‐to‐severe)	5 (71.43)	‐	‐	‐	‐	‐	‐	‐	‐	‐
	AF with CMBs burden	229 (69.18)	1.04	0.81–1.33	0.77	1.05	0.82–1.36	0.68	1.09	0.85–1.40	0.51
	AF with presence of CMBs	74 (74.75)	1.39	0.87–2.23	0.17	1.40	0.87–2.24	0.16	1.42	0.88–2.27	0.15
	AF with CSVD (Wardlaw)	79 (70.54)	1.02	0.66–1.57	0.94	1.03	0.67–1.59	0.89	1.08	0.70–1.67	0.73
	AF with CSVD (Rothwell)	70 (70.00)	0.98	0.62–1.54	0.93	1.00	0.64–1.58	0.99	1.02	0.65–1.61	0.92

*Note*: Model 1: adjusted for age, sex, and NIHSS; Model 2: adjusted for age, sex, NIHSS, and mRS; Model 3: adjusted for age, sex, NIHSS, mRS, and risk factors (hypertension, diabetes mellitus, dyslipidemia, previous ischemic stroke or TIA). WMH burden was defined as either (early) confluent deep white matter hyperintensities (Fazekas score 2 or 3) or irregular periventricular white matter hyperintensities extending into the deep white matter (Fazekas score 3); modified WMH burden was classified into grade 0: total periventricular + subcortical white matter hyperintensities score 1–2, grade 1: total periventricular + subcortical white matter hyperintensities score 3–4 and grade 2: total periventricular + subcortical white matter hyperintensities score 5–6. The presence of CMBs was defined as the presence of cerebral microbleeds; CMBs burden was classified as grade 0: absent, grade 1: 1–4 microbleeds, and grade 2: ≥5 microbleeds. BG‐PVS (moderate‐to‐severe) indicated >10 perivascular space in basal ganglia.

Abbreviations: AF, atrial fibrillation; CMBs, cerebral microbleeds; CSVD, cerebral small vessel disease; PVS, perivascular space; WMH, white matter hyperintensity.

The results showed that ischemic stroke patients with both AF and CSVD burden (Wardlaw) had an increased risk of all‐cause mortality (HR 5.10, 95% CI 2.32–11.20, *p* < 0.001), at 12‐month follow‐up compared to patients without AF and CSVD burden (Wardlaw). However, no significant increase in the risk of stroke recurrence or poor functional outcome at 12‐month follow‐up was observed in ischemic stroke patients with both AF and CSVD burden (Wardlaw) (Table 4a). Similarly, compared to patients without AF and CSVD burden (Rothwell), ischemic stroke patients with both AF and CSVD (Rothwell) had an increased risk of stroke recurrence (HR 1.82, 95% CI 1.03–3.20, *p* = 0.04) at 12‐month follow‐up. However, no significant increase in the risk of all‐cause mortality or the poor functional outcome at 12‐month follow‐up was observed in ischemic stroke patients with both AF and CSVD burden (Rothwell) (Table 4b).

**TABLE 4:** (a) The association between atrial fibrillation, CSVD (Wardlaw), and 12‐month outcomes and (b) the association between atrial fibrillation, CSVD (Rothwell) and 12‐month outcomes.

	Atrial fibrillation and CSVD	Events (%)	HR/OR	95% CI	*p*
(a)					
Stroke recurrence	No AF without CSVD (Wardlaw)	149 (8.44)	Ref.	Ref.	Ref.
	No AF with CSVD (Wardlaw)	138 (8.30)	0.95	0.75–1.20	0.67
	AF without CSVD (Wardlaw)	13 (10.24)	1.09	0.61–1.94	0.76
	AF with CSVD (Wardlaw)	17 (15.18)	1.63	0.96–2.76	0.07
All‐cause mortality	No AF without CSVD (Wardlaw)	19 (1.08)	Ref.	Ref.	Ref.
	No AF with CSVD (Wardlaw)	31 (1.86)	1.43	0.80–2.56	0.23
	AF without CSVD (Wardlaw)	5 (3.94)	‐	‐	‐
	AF with CSVD (Wardlaw)	13 (11.61)	5.10	2.32–11.20	<0.001
Poor functional outcome	No AF without CSVD (Wardlaw)	971 (54.98)	Ref.	Ref.	Ref.
	No AF with CSVD (Wardlaw)	955 (57.43)	0.95	0.82–1.10	0.51
	AF without CSVD (Wardlaw)	75 (59.06)	0.85	0.57–1.25	0.40
	AF with CSVD (Wardlaw)	79 (70.54)	1.13	0.72–1.77	0.60
(b)					
Stroke recurrence	No AF without CSVD (Rothwell)	123 (7.55)	Ref.	Ref.	Ref.
	No AF with CSVD (Rothwell)	135 (8.88)	1.15	0.90–1.48	0.26
	AF without CSVD (Rothwell)	10 (8.62)	1.03	0.54–1.99	0.93
	AF with CSVD (Rothwell)	15 (15.00)	1.82	1.03–3.20	0.04
All‐cause mortality	No AF without CSVD (Rothwell)	17 (1.04)	Ref.	Ref.	Ref.
	No AF with CSVD (Rothwell)	31 (2.04)	1.61	0.88–2.94	0.12
	AF without CSVD (Rothwell)	5 (4.31)	‐	‐	‐
	AF with CSVD (Rothwell)	4 (4.40)	‐	‐	‐
Poor functional outcome	No AF without CSVD (Rothwell)	885 (54.33)	Ref.	Ref.	Ref.
	No AF with CSVD (Rothwell)	854 (56.15)	0.90	0.78–1.05	0.19
	AF without CSVD (Rothwell)	72 (62.07)	0.97	0.64–1.47	0.89
	AF with CSVD (Rothwell)	70 (70.70)	1.07	0.67–1.72	0.78

*Note*: The events refer to the occurrences of stroke recurrence, all‐cause mortality, and poor functional outcomes. Adjusted for age, sex, NIHSS, mRS, and risk factors (hypertension, diabetes mellitus, dyslipidemia, previous ischemic stroke or TIA).

Abbreviations: AF, atrial fibrillation; CSVD, cerebral small vessel disease.

## 4 DISCUSSION

In this study, the patients who have AF and CSVD imaging were associated with a higher risk of poor prognosis. However, previous studies have shown a link between AF and WMH; the associations between AF and PVS or lacunes were not clear.^23^ And there have been studies reporting that in individuals without a history of stroke, there was no definite association between AF and WMH. When subgrouping AF patients, whether they received anticoagulation therapy could also influence the impact of AF on WMH.^6^ From a genetic background perspective, there may be a genetic basis because studies have found a genetic correlation between AF, stroke, and WMH. These genes may be involved in the development and progression of both AF and cerebral pathology.^24^

Regarding the prognosis of AF and ischemic stroke, some research conclusions have been made. For example, nonparoxysmal atrial fibrillation patients had a higher risk of poor prognosis. However, no differences in prognosis were found between the group with atrial fibrillation diagnosed after stroke and the group with atrial fibrillation known before stroke.^25^ Research on the prognosis of AF, CSVD, and ischemic stroke was of great clinical significance. Theoretically, ischemic strokes associated with AF may be more severe or have larger lesions, with a higher probability of poor prognosis. Additionally, the imaging features and different combinations of CSVD were closely associated with ischemic and hemorrhagic stroke, as well as all‐cause mortality.^26^, ^27^ Previous clinical studies have suggested that ischemic stroke patients with concomitant CSVD on imaging have an increased risk of bleeding and disability at 3 months.^28^ The CSVD burden and the number of infarctions could predict the risk of stroke recurrence in patients with acute minor stroke and transient ischemic attack during the 1‐year follow‐up.^29^

Recent studies showed that WMH could predict the neurological functional status of patients after stroke. The higher the Fazekas score of the patients, the worse their neurological functional prognosis and mRS at 3 months.^30^, ^31^ And the brainstem WMH on imaging was associated with an increased risk of unfavorable mRS at 90 days after ischemic stroke.^32^ And the patients with ischemic stroke with the burden of CSVD and lacunes were associated with thrombotic recurrence.^33^ Having more than 20 PVSs in the basal ganglia region was associated with an increased risk of recurrent ischemic stroke.^34^ Previous studies have evaluated the relationship between CMBs on imaging and outcomes in patients with nonvalvular AF receiving oral anticoagulation therapy. The results suggested that there was no association between CMBs and recurrent ischemic stroke. However, the presence of CMBs on MRI and ≥5 CMBs could help identify individuals at high risk of bleeding.^35^ In another study, patients receiving warfarin anticoagulation were evaluated for both imaging and clinical outcomes. Follow‐up revealed that patients with 0 to 4 CMBs had a lower incidence of intracranial hemorrhage (ICH) compared to ischemic stroke. However, patients with ≥5 CMBs had a higher incidence of ICH. Additionally, CMB count was even more sensitive than the CHA2DS2‐VASc score in predicting ICH. In another study, patients receiving warfarin anticoagulation were evaluated for both imaging and clinical outcomes. Follow‐up revealed that patients with 0 to 4 CMBs had a lower incidence of ICH compared to ischemic stroke. However, patients with ≥5 CMBs had a higher incidence of ICH. Additionally, CMB count was even more sensitive than the CHA2DS2‐VASc score in predicting ICH.^36^

This study suggested that when AF was combined with CSVD such as lacunes, WMH, and presence of CMBs, there was an increased risk of poor prognosis at 12 months. In addition, the association between AF and the burden of CSVD suggested an increased risk of poor prognosis at 12 months. This was consistent with previous findings that the burden of stroke‐related disease was relatively heavy in AF, and it could lead to chronic cerebral hypoperfusion.^37^ The mechanism of endothelial damage, brain barrier disruption, and inflammation associated with combined with the association between these two diseases, significantly increased the risk of stroke recurrence, bleeding, and death. Therefore, for ischemic stroke patients with AF, it was also important to pay attention to MRI findings of CSVD. Patients with imaging features such as lacunes, WMH, and CMBs may require more careful long‐term management of risk factors, as well as a balanced approach to treating the stroke and CSVD.

The strengths of this study included exploring the associations among AF, CSVD, and 12‐month outcomes of ischemic stroke in a large, representative cohort of patients. However, there were also limitations to this study. First, the population was based on Chinese individuals, when generalizing the findings to other racial or ethnic groups, the conclusion should be caution. Second, although we found associations, further research was needed to determine the specific relationships and underlying pathophysiological mechanisms. Lastly, some groups had a small sample size after multiple stratifications, such as variables related to PVS, and further validation and research can be conducted in populations with larger sample sizes.

## 5 CONCLUSION

In the Chinese population with acute ischemic stroke, atrial fibrillation was also found to be associated with higher 12‐month mortality and stroke recurrence in stroke patients. Further investigation revealed that when atrial fibrillation was combined with cerebral small vessel disease imaging and burdens in stroke patients, the 12‐month prognosis including death, stroke recurrence, and poor functional outcome worsened.

## AUTHOR CONTRIBUTIONS

YlW took responsibility for analysis. YcW, YP, HL, XL, MW, and YlW contributed to study concept and design. YP, MW, XM, WC, and YY were involved in acquisition, analysis, or interpretation of data. YcW carried out drafting of the manuscript. YP and MW carried out statistical analysis and study supervision. YlW and YoW obtained funding.

## FUNDING INFORMATION

This study was supported by grants from the National Natural Science Foundation of China (No. 81825007); Beijing Outstanding Young Scientist Program (No. BJJWZYJH01201910025030); Youth Beijing Scholar Program (No.010); Beijing Talent Project – Class A: Innovation and Development (No. 2018A12); “National Ten‐Thousand Talent Plan” – Leadership of Scientific and Technological Innovation; Chinese Academy of Medical Sciences Innovation Fund for Medical Sciences (2019‐I2M‐5‐029).

## CONFLICT OF INTEREST STATEMENT

The authors declare no conflicts of interest.
